# Frailty assessment and candidate optimization before cardiothoracic transplantation

**DOI:** 10.1016/j.jhlto.2026.100492

**Published:** 2026-01-17

**Authors:** Antoine Premachandra, Jacob Klapper, Jonathan Messika, Maira Gaillard, Martin Kloeckner, Kamrouz Ghadimi, Matthieu Reffienna, Krista Ingle, Kirti Magudia, Brandi Bottiger

**Affiliations:** a Department of Anesthesiology, Foch Hospital, Suresnes, France; b Department of Anesthesiology, Marie Lannelongue Hospital, Le Plessis-Robinson, France; c Division of Cardiothoracic Surgery, Department of Surgery, Duke Hospital, Durham, NC; d Thoracic Intensive Care Unit, Lung Surgery and Transplantation, Foch Hospital, Suresnes, France; e Department of Cardiac Surgery, Marie Lannelongue Hospital, Le Plessis-Robinson, France; f Department of Cardiology, Marie Lannelongue Hospital, Le Plessis-Robinson, France; g Cardiothoracic Anesthesiology and Critical Care Medicine Divisions, Department of Anesthesiology, Duke Hospital, Durham, NC; h Department of Physical Therapy, Foch Hospital, Suresnes, France; i Department of Psychiatry and Behavioral Sciences, Duke Hospital, Durham, NC; j Department of Radiology, Duke Hospital, Durham, NC

**Keywords:** Frailty, Heart transplantation, Lung transplantation, Prehabilitation, Cachexia, Sarcopenia, Biomarkers

## Abstract

Frailty is an important syndrome in an aging thoracic transplant population, particularly in the context of end-stage cardiopulmonary disease. In this review, we discuss the core problem of lack of physiologic reserve in these patients and its relationship to perioperative outcomes, how to define and measure frailty in the context of heart and lung transplant and identify interventions to treat contributors to this clinical syndrome. We highlight the critical need in the transplant community to identify and treat these conditions prior to their transplantation and to prevent comorbidity and mortality.

## Background

Frailty, defined as a state of heightened vulnerability and reduced physiological reserve, is a key risk factor for poor perioperative outcomes after cardiothoracic transplantation, regardless of age. Compared to non-transplant peers, the incidence of frailty among heart and lung transplant recipients is extremely high. Frailty affects 20%-40% of LTx candidates and ∼40% of HTx candidates, versus 4%-16% in other surgical patients.^1^, ^2^

Patients with pre-transplant frailty have a marked increase in the risk of morbidity and mortality. In one recent meta-analysis of 1.1 million surgical patients in 56 studies, frailty associated with higher 30-day mortality and complication rates.^3^ In cardiothoracic transplant candidates, frailty is linked to elevated risks of waitlist mortality, infections, multiorgan dysfunction, and prolonged hospital stay. Recent consensus guidelines emphasize the need to assess and optimize frailty, advocating for multidisciplinary evaluation and incorporation into listing decisions.^4^, ^5^, ^6^ However, standardizing frailty measurement remains challenging due to heterogeneity in phenotypes, assessment tools, timing, and limitations in physical performance testing in end-stage cardiopulmonary patients. These factors make it nearly impossible to impose strict thresholds for listing or transplant.

Early identification of frailty allows clinicians to provide resources to patients and set up targeted interventions. Prehabilitation programs multimodal in nature, usually aimed at physical conditioning, nutrition, patient education, and coping strategies prior to surgery to prevent further decline and optimize transplant outcomes. These programs are both feasible and beneficial through stabilizing or improving exercise capacity and health-related quality of life while awaiting transplant.^7^

The concept of reversing frailty remains controversial and is not guaranteed after transplant surgery. Several studies describe 2 phenotypes of frailty: age-related and disease-related,^8^ of which disease-related is more likely to be reversible. The concept of frailty reversal through transplantation is more tangible when modifiable conditions outweigh non-modifiable conditions, rather than depending on the ability of providers to make a strict binary distinction between “primary” and “secondary” frailty. Because these categories often coexist, and it is often difficult to distinguish between them in this patient subgroup. This paradigm describes improving and declining clinical trajectories after the time of organ transplant or bridging therapy. This is also relevant when considering surgical interventions for the advanced heart failure (HF) patient awaiting transplant, which has been revolutionized through bridging with mechanical circulatory support. This is often viewed as an opportunity to stabilize or improve cardiopulmonary failure and therefore improve secondary frailty. Examples include peripherally cannulated extracorporeal membrane oxygenation, temporary or durable left ventricular assist devices or total artificial heart, which improve perfusion.

This is a thematic narrative review aiming to synthesize and contextualize current knowledge on frailty in cardiothoracic transplant candidates, with a focus on contributors, measurement, and optimization strategies. We conducted a non-systematic literature search in PubMed using combinations of the terms “frailty,” “heart transplantation,” “lung transplantation,” “sarcopenia,” “cachexia,” “rehabilitation,” “prehabilitation,” “phenotype,” and “biomarkers.” Articles were included if they were judged relevant by the authors to address physical, cognitive, nutritional, psychosocial, or biological dimensions of frailty. The review was considered sufficiently comprehensive once successive searches yielded no new major themes. We first examine key concepts and definitions in frailty, and those that specifically apply to the thoracic transplantation population. Secondly, we propose prehabilitation strategies that target modifiable components of frailty in transplant candidates before surgery.

## Frailty syndrome

### Frailty concept

**Figure 1:**
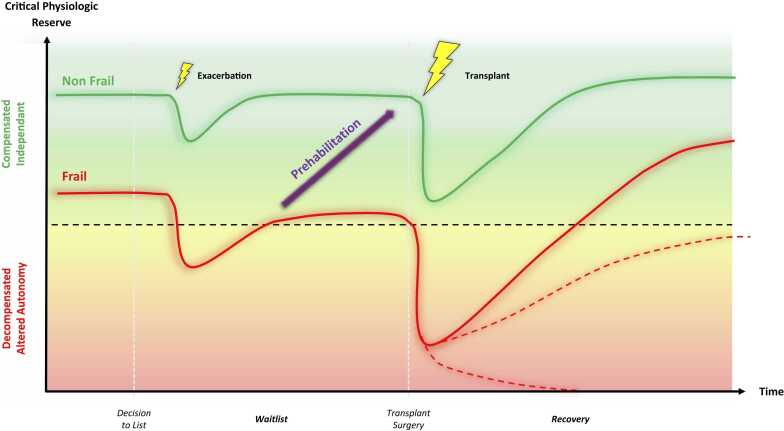
*Clinical trajectories of frail and nonfrail patients in response to stressors.* Frail patients (red line) have extremely limited physiologic reserve and may not be able to recover from a physiologic insult (e.g., disease exacerbation, major transplant surgery) while non-frail patients may tolerate the physiologic insult and can recover without decompensating into critical illness (green line). The dotted lines represent the uncertainty in the prognosis of frail patients, which can vary according to the type of frailty (mainly disease-related versus age- or comorbidity-dependent) from deterioration leading to death to marked improvement. Prehabilitation or treatment of secondary frailty may theoretically allow for recuperation of physiologic reserve, facilitating recovery (purple arrow). Adapted from “Frailty in the End-Stage Lung Disease or Heart Failure Patient,” Bottiger et al, J Cardiothorac Vasc Anesth, Volume 33, Issue 5, 1382-1392, 2019 May, with permission.

The patient with frailty has very little physiologic reserve to withstand stressors, where subclinical dysfunction is present at baseline. Any clinical insult in a frail patient can cause rapid deterioration and become the inciting event for critical illness, decompensation and comorbid perioperative course (Figure 1). Although distinguishing whether frailty is due to an underlying condition independent of cardiopulmonary disease (primary frailty) or secondary to end stage cardiopulmonary disease (secondary frailty) may be heuristically useful,^8^ a strict a priori classification is rarely feasible in this setting. Instead, a deeper understanding of the main contributors to frailty is essential before selecting potential interventions. The goal is to identify patients whose frailty may improve with bridging therapies, enabling them to recover physiologic reserve to some extent and achieve a meaningful quality of life after surgical intervention.

### Functional frailty - pathophysiology and contributors

Functional frailty refers to the clinically observable decline in physical performance, such as reduced gait speed, impaired mobility, or diminished muscle strength, captured by bedside functional tests. This contrasts with other domains of frailty (e.g., cognitive) and emphasizes the measurable, performance-based component of the syndrome.

#### Sarcopenia and cachexia

Reduced muscle mass and diminished strength or function of at least one peripheral muscle indicates age or disease-related *sarcopenia*.^9^ Body composition and muscle mass may be directly measured by cross sectional area through radiologic imaging such as computed tomography (CT) or magnetic resonance imaging, bioelectrical impedance, or measurement of skin folds. Isometric and isokinetic testing are useful measurements of muscle strength of the upper and lower extremities. Muscle ultrasound is an alternative, validated, reproducible, and non-invasive method that can be easily performed at the bedside to assess both the quantity of muscle (via cross-sectional area or layer thickness) and its quality (e.g., echogenicity). Unlike CT or magnetic resonance imaging, ultrasound enables close and repeated monitoring of muscle changes before and after transplantation, making it a practical tool to detect sarcopenia and track its trajectory over time.^10^ *Cachexia,* or body wasting, is measured by a combination of clinical symptoms and the presence of several inflammatory biomarkers. Clinical signs include an unintentional weight loss of 5% or more of body weight over 6 months with one of the following: decrease in muscle strength, fatigue, anorexia, low fat-free mass index (defined as mid-upper arm muscle circumference <10th percentile for age and gender). Biomarkers associated with cachexia include increased CRP or IL-6, reduced hemoglobin, and low serum albumin.^11^ Both sarcopenia and cachexia reduce quality of life, worsen surgical outcomes, and predict perioperative morbidity and mortality.^11^, ^12^

#### Contributors to sarcopenia

Poor nutritional status contributes to the states of sarcopenia and cachexia, which can be challenging to overcome in a patient with end-stage cardiopulmonary failure. Optimization of nutrition is a critical element of many surgical prehabilitation programs, with transplant specific-issues discussed below. Nutrition optimization with exercise training, anabolic agents (e.g., appetite stimulants, testosterone supplementation), and anti-inflammatory agents can modify frailty secondary to HF.^13^ Beyond nutrition, sarcopenia in advanced cardiopulmonary disease is influenced by age-related changes in muscle biology (oxidative stress, myosteatosis), endocrine alterations (insulin resistance, hypogonadism), systemic inflammation, immobility and deconditioning, as well as by certain drugs and comorbidities.

Assessing nutritional status pre- and post-transplant is crucial, as malnutrition accelerates disease progression and impairs function. Regular nutritional evaluations and dietary counseling both before and after transplant are essential. The European Society for Clinical Nutrition and Metabolism defines severe nutritional risk as BMI < 18.5 kg/m², >10% weight loss in 6 months, a Nutritional Risk Score > 5, or serum albumin < 30 g/L (excluding hepatic or renal disease). Malnutrition is classified by BMI < 18.5 kg/m² or a combination of ≥5% weight loss in 3 months or ≥10% in 6 months with BMI <20 kg/m², or low fat-free mass index.^14^

It is also well established that malnutrition and sarcopenia may also coexist with obesity (“sarcopenic obesity”). Adipose tissue disrupts the inflammatory cytokine balance through production of adipokines, such as leptin and adiponectin, which results in an overall state of systemic inflammation. Further, leptin has been associated with the development of acute lung injury and severe primary graft dysfunction after LTx.^15^

#### Measurement tools for assessment of frailty

##### Clinical tools

The core components of commonly used frailty quantification tools for transplant patients include measures of physical function, mobility, nutritional status, strength, energy, cognition, mood, and social support. Of the more than 20 frailty quantification tools that have been described, few have demonstrated convergent predictive validity for both medical and surgical patients: Rockwood’s Frailty Index,^16^ the Fried Frailty Phenotype (FFP),^17^ and the Clinical Frailty Scale (CFS), with other assessment tools shown in Table 1. Rockwood’s frailty index is focused on the measurement of deficit accumulation, where a lifetime of molecular damage results in organ dysfunction and clinical presentation of frailty. In contrast, the FFP describes frailty by the clinical assessments of sarcopenia, muscle strength, and physical exertion. The CFS is a questionnaire that quantifies the amount of support needed in physical, social, and cognitive domains, allowing for a 9-category ranking of frailty. The CFS outperformed the FFP and Frailty index for predicting death and disability in large cohorts of surgical patients. For thoracic transplant patients, the FFP coupled with the short physical performance battery (SPPB) has shown to be a very valuable measure of frailty. Here, measurement of functional status is emphasized, with poor performance being associated with mortality and postoperative complications after LTx and increased risk of delisting or death. By assessing exercise capacity, the 6-minute walk test (6MWT; i.e., the distance walked in 6 min) is the most studied functional measure for post-LTx prognosis, whereas evidence for other physical function and frailty assessments remains more limited.^18^ Singer et al. offer the Lung Transplant Frailty Scale (LT-FS)*,* which outperforms the SPPB and the FFP in predicting delisting, waitlist mortality, and 1-year mortality (area under the curve for waitlist death or delisting: LT-FS 0.73, SPPB 0.58, FFP 0.55).^19^ The LT-FS integrates the balance and gait speed components from the SPPB, the grip strength component from the FFP, and CRP levels. Similar combinations of clinical measures, body composition, and biomarkers of inflammation have been proposed for HTx candidates as well (See “Future Directions”).

**Table 1:** Frailty Assessment Tools as Reported by Thompson et al^5^

Name of tool	Items measured	Score
Fried frailty phenotype^17^	One point each for Slowness, low activity, weight loss, exhaustion, weakness	0 = Nonfrail, 1-2 = Prefrail, 3-5 = Frail
Rockwood Deficit Accumulation Index^16^	20-70 deficits measured across domains	Number of deficits/Higher scores indicate greater frailty
Edmonton Frail Scale^20^	10 deficits across domains	Sum of scores/17
		Higher scores indicate greater frailty
FRAIL scale^21^	One point each for stair climb, ambulation, fatigue, illnesses >5, weight loss >5%	0 = Nonfrail, 1-2 = Intermediate, 3-5 = Frail
SPPB^16^	Gait speed, chair stands, balance tests	Maximum 4 points per item, range, 0-12 points.
		>10 = Nonfrail, 3-9 = Frail, <2 = disabled
Clinical Frailty Scale (CFS)^22^	Clinician assessment results in scoring in 9 categories ranging from very fit to terminally ill	Categories 5-8 indicate mild, moderate, severe and very severe frailty
LT-FS^19^	Previously established measures of SPPB and CFS plus routine labs, body composition, and biomarker panel (http://lungtransplantfrailtyscale.ucsf.edu)	Produced 3 models with proposed “cut points” for frail vs not frail
HFFS^23^	Clinical, psycho-cognitive, functional, social domains	Not validated yet

Abbreviations: HFFS, heart failure frailty score; LT-FS, lung transplantation frailty scale; SPPB, short physical performance battery.

The challenges in implementing generalized and established frailty measurement in this setting is that they rely heavily on physical performance testing, and often these patients may be limited by dyspnea related to HF, hypercapnia, or hypoxemia. In HF especially, weight loss may not be a good reflection of muscle mass and adiposity but may include a significant amount of weight related to tissue edema. Therefore, specific frailty measures that are objective, reproducible, and independent of cardiopulmonary failure are needed. In isolated pulmonary failure, dyspnea related to hypoxemia can be somewhat overcome by supplemental oxygen but relies on a well-established pulmonary rehab facility for monitoring and supervised exercise to truly test physical limits.

##### Biomarkers

**Figure 2:**
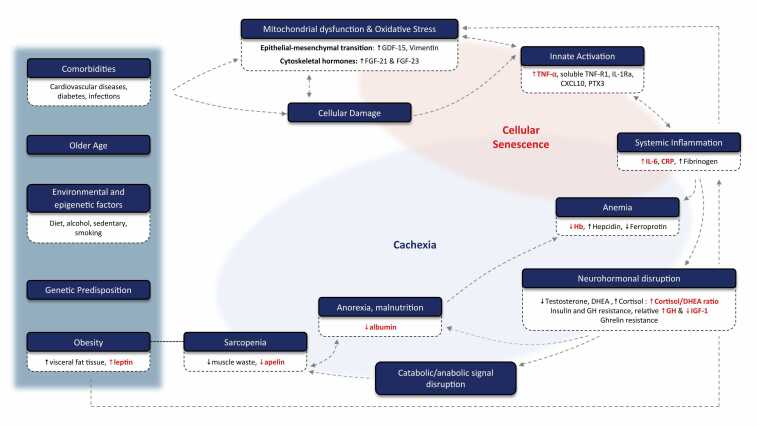
*Biomarker changes associated with physical frailty.* The clinical risk factors for physical frailty are listed on the left, while biomarkers are based on the pathobiological cascade mapped in the figure. Markers of inflammation seem to be the most relevant to mortality risk, with IL-6 and CRP being consistent between LTx and HTx populations. Persistent inflammation fuels cellular senescence, then upends neurohormonal balance (relative GH excess, low IGF-1, insulin and GH resistance, ↑ cortisol/DHEA). This catabolic state drives muscle wasting (sarcopenia), blunted ventilatory response, exercise intolerance, and chronotropic incompetence that contribute to cachexia. CXCL10: interferon-inducible protein-10, DHEA: dehydroepiandrosterone, FGF-21/23: Fibroblast growth factor 21/23, GDF-15: Growth differentiation factor 15, GH: growth hormone, IGF-1: insulin-like growth factor-1, IL-1Ra: Interleukin-1 receptor antagonist, IL-6: interleukin 6, TNF-α: tumor necrosis factor, TNF-R1: TNF receptor-1.

Both primary and secondary frailty from cardiopulmonary failure show similar patterns of cellular senescence and injury to include mitochondrial dysfunction, oxidative stress, subclinical systemic inflammation, and neurohormonal dysregulation. The ultimate result is a chronically stressed and catabolic state with detrimental systemic effects.^8^ This serves not only as a potential way to measure the disease of frailty and sarcopenia but may represent future therapeutic targets. Sources of innate inflammation found in frailty stem both from an age-related decline in immune function and from senescent-associated secretory cells, which release proinflammatory cytokines, chemokines, and proteases.^24^ The biomarkers are based on the pathobiology of frailty and are shown in Figure 2. The most relevant in heart and lung failure populations seem to be the biomarkers of inflammation. In one multicenter pilot study from the LTBC group, 422 LTx patients were identified as having clinical frailty based on performance on SPPB < 12, measured body composition, and serum biomarkers. The study team used latent class analysis to measure subgroups of frail patients, which produced 2 phenotypes of frailty, those with and without systemic innate inflammation. Those with frailty and systemic innate inflammation had higher risk of waitlist delisting or death (HR 4.0, 95% CI [1.8-9.1]). Patients who had both sarcopenia and systemic innate inflammation had higher inflammatory proteins (IL-6, CRP, PTX3, TNF-R1, IL-1Ra), elevated markers of mitochondrial stress (GDF-15, FGF-21), sarcopenia, malnutrition, lower hemoglobin, and shorter walk distance.^25^

### Cognitive frailty

Although frailty is classically described as being measures of body composition and physical performance, there are various dimensions of cognition, psychiatry, and social support that must be considered for a comprehensive assessment. The concept of cognitive frailty is characterized by the simultaneous presence of both physical and cognitive impairment.^26^ HF patients are at particular risk, as they experience a higher incidence of cognitive impairment and depression than non-HF populations. Subtypes of frailty within the HF population include physical frailty alone, depressive frailty (physical frailty and depression), cognitive frailty (cognitive impairment and physical frailty), and cognitive-depressive frailty (physical frailty, depression, and cognitive impairment). Reduced 1-year survival was found among frail patients that underwent HTx (52% ± 23 vs 100%) or VAD as bridge- to- transplant (58% ± 15 vs 71% ± 14).^27^ In pursuit of better risk stratification, additional concepts warrant consideration. Low grit (perseverance and passion for long-term goals, evaluated by the 12-Item Grit Scale) and resilience (ability to maintain stable psychological and physical functioning after a stressful event, evaluated by the Brief Resilience Scale) have also been associated with worse early post-transplant outcomes.^28^

## Addressing frailty

Targeting improvement in the frail condition prior to transplantation surgery relies on identifying modifiable drivers of frailty through specific intervention, ideally performed in enough time to assess the improvement. A major goal of candidate optimization is to reduce the risk of postoperative frailty (i.e., new or worsened functional decline, malnutrition, or deconditioning) by anticipating conditions that can precipitate vulnerability after surgery. Supporting the cognitive, behavioral, and social elements of the patient and provide long-term strategies for optimization is equally important. Transplant prehabilitation programs that encompass the physical, nutritional, social, and mental health and focus on optimization of medical comorbidities are therefore essential to improve physiologic reserve if possible and reduce the patient’s vulnerability to the stress of surgery.

### Addressing the frailty syndrome in recipients

#### Prehabilitation programs

Prehabilitation involves multimodal interventions that typically integrates physical training, psychological support, and nutritional interventions, while acknowledging the interdependence of physical, mental, and nutritional health. Significant challenges include mobilizing these patients in a monitored setting and ensuring adequate preoperative protein caloric intake in the setting of cachexia. Physical training improves cardiovascular function and muscle strength, psychological support helps manage depression and anxiety, and fosters positive coping strategies, and nutritional interventions optimize body composition and immune function. Some muscle loss during the hospital stay is to be expected, so it is important that patients are at their physical best. Following transplant, patients experience significant muscle catabolism while in the intensive care unit related to post-operative inflammation and high-dose corticosteroids, with muscle mass loss reaching up to 2% per day and up to 30% of total muscle mass after LTx.

Publications surrounding the effectiveness of prehabilitation programs most often compare historical cohorts before and after implementation. Recently, Appiani et al. demonstrated in a study of 12 hospitalized HTx-LTx candidates (compared to 12 patients in a historical cohort) that their prehabilitation program was associated with earlier extubation and improved post-transplant muscle function.^29^ Similarly, Lopez-Baamonde et al evaluated 31 outpatient HTx candidates (compared to 51 in a historical cohort) and found that their program led to a reduction in postoperative complications, mechanical ventilation duration, ICU and hospital length of stay, and a greater proportion of patients able to return home, all without increasing costs.^7^ Furthermore, the recent Enhanced Recovery after Surgery and Society of Thoracic Surgeons 2024 Expert Consensus Statement on Perioperative Care focus on the 3-part model for prehabilitation, including physical exercise, nutritional deficiency correction, and psychological/cognitive assessment and counseling.^30^

##### Physical exercise

For HTx-LTx candidates, exercise is often challenging due to breathlessness and hypoxemia associated with their primary disease. However, exercise training is both feasible and well tolerated in this population with proper monitoring and support.^31^ For patients with HF, aerobic exercise training for patients is a class I recommendation by the European Society of Cardiology guidelines, the American college of Cardiology, and the American Heart Association.^32^, ^33^ Maintaining or increasing skeletal muscle mass during evaluation and the waiting period is probably the most important reversible target for improving postoperative recovery, and could translate into shorter ICU and hospital stay as well as reduced duration of mechanical ventilation.^34^ Moreover, prehabilitation can enhance the likelihood of early adherence to a rehabilitation program starting in the immediate postoperative intensive care phase. While there is no consensus on the optimal training modalities, *the type of recommended exercise intervention* includes 1) aerobic training to enhance cardiovascular function, 2) strength training to build muscle mass, 3) inspiratory muscle training to improve respiratory strength and efficiency, or a combination of these approaches. *The training load* is generally based on an *initial clinical assessment*, such as the dyspnea and the oxygen needed during a 6MWT for aerobic training or the initial medical research council scale for strengthening training. *The training regimen* typically consists of 1-hour sessions, monitored at a minimum by SpO_2_, with a *frequency* of 2-6 sessions per week. While these protocols are primarily hospital-based, involving in-person sessions with a physical therapist and continuous monitoring, recent work by Bourgeois et al. demonstrated that a *virtual* prehabilitation program with home-based sessions conducted via teleconference with a physical therapist was both safe and effective in 20 LTx candidates.^35^ This approach allowed patients to improve or maintain their status, limb strength, functional exercise capacity, and quality of life. Table 2 provides 3 specific examples of these exercise protocols.

**Table 2:** Successful Monitored Rehabilitation Protocols That Demonstrated Improvement of Specific Components of Frailty (Physical, Nutritional, Psychological)

Study		Appiani et al (*n* = 12 vs 12)	Lopez-Baamonde et al (*n* = 31 vs 51)	Bourgeois et al (*n* = 20)
Patient population		Heart or lung transplant candidates	Heart transplant candidates	Lung transplant candidate
		Hospitalized patients only	Outpatients: induction phase in hospital, maintenance phase at home	Outpatients: induction phase at home (supervised), maintenance phase at home (independent)
Physical	Assessment tool	*Exercise tolerance* using the Borg scale during a 6MWT and a Sit-to-Stand Test. *Muscle strength* using the MRC scale	Functional capacity evaluation using the peak work rate (pWR) during an incremental exercise on a cycle ergometer.	O_2_ needed during the 6MWT.
	Frequency	Six sessions of 30-60 min/week, alternating between aerobic and strength training	Two sessions of 60 min/week, combining high-intensity interval training, strength training and breathing exercise	
	Aerobic training	Continuous loading, constant speed 15 km/h *1 cycle*: 1-3 min, then 1-3 min of rest This cycle is repeated for 30-60 min *Intensity*: 50%-60% of the initial Borg, then modulated based on the catabolic state. *Monitoring*: SpO_2_, oxygen for >90%	High-intensity interval training on a stationary bike 5′ warm-up + 5′ cool-down at 30% pWR. Interval training with 5 rounds combining - 2′ of high-intensity exercise (starting at 70% pWR and progressing to 90%-100% pWR) - interspersed with 3′ of low-intensity recovery periods (40%-50% pWR). *Monitoring*: EKG, SpO_2_, NIBP, dyspnea	Independent aerobic exercises: 5 times/week. Fixed O_2_ therapy based on the O_2_ needed in the initial 6MWT. *Monitoring*: SpO_2_ at home, stop if <85%.
	Strength training	*Upper extremity muscle*: Pull, Push, and Flexion and Extension of the elbow. *Lower extremity muscle:* Squats, Knees Flexion & Extension, Ankle Plantar Flexion Each exercise: 3 sets of 6-15 repetitions (stop if SpO_2_ <90% or HR >120 bpm) Load adjusted on the Nitrogen Balance	Upper-limb and core muscle exercise avoiding Valsalva maneuvers. 6 to 12 repetitions (based on muscular exhaustion)	Supervised lower and upper body strengthening: 3 videoconferences/week - Functional exercises for lower extremities - Weight exercises for upper extremities with existing home equipment such as dumbbells, elastics or bottles/cans (no exercise equipment given to patients). - Intensity: 3-4 on Borg Scale
	Respiratory training		Breathing exercise: with an incentive spirometer	
	Duration	Until the transplant	8 weeks	12 weeks
	Maintenance		1 session/w *supervised* exercise training Promotion of home-based exercising	12 weeks with *independent* home exercises
Nutritional	Goals	- Energy: 35-40 kcal/kg/d if underweight 12-15 kcal/kg/d if obesity - Protein: usually 1.5-2 g/kg/d, adapted on pathology, training, and nitrogen balance - Carbohydrates: 45% of the diet in 4-6 meals - Lipids: 30% of the diet - Vitamins and trace elements as needed Route: oral/nasoenteral tube/gastrostomy as needed	Based on the 2017 EPSEN Guidelines on clinical nutrition in surgery. + Whey protein supplementation within 1 hour after training and before sleep to achieve a daily protein intake of 1.5-2 g/kg.	
Psycho-logical		Psychological sessions 1-3 times each week +/- other interventions such as crisis interventions, problem-solving, psychoeducation, stress reduction techniques (stress management and relaxation techniques), group therapy and work with family members or caregivers	Mindfulness group session 1 session/w, recommended especially in patient with anxiety/depression HADS score > 8 + Group session of breathing and relaxation exercises 1 time each week.	

Abbreviation: MRC scale, medical research council scale; MWT, minute walk test.

A fully digital prehabilitation program,^36^ delivered via a mobile app with personalized exercise plans, instructional videos, and nutrition plan, was shown to be safe and reduced frailty scores. However, it did not improve 6MWT, probably because of modest adherence (∼60%), highlighting that digital tools may expand access but cannot fully replace in-person support for patients with comorbidities or low engagement.

##### Nutrition

Perioperative nutritional support is recommended for malnourished candidates or those considered high nutritional risk. If adequate intake (25-30 kcal/kg and 1.5 g protein/kg) cannot be met orally, enteral nutrition should be considered through nasogastric or gastrostomy tube.^14^ Metabolic conditioning aims to prevent insulin resistance, which contributes to post-transplant complications. Preoperative carbohydrate loading reduces insulin resistance, hypoglycemia, and stress responses, prompting European Society for Clinical Nutrition and Metabolism to recommend oral carbohydrate intake instead of fasting in general surgery (800 mL the night before and 400 mL 2 hours before surgery). However, this approach has not been thoroughly assessed in the context of transplant and is not mentioned in transplant specific guidelines.^37^, ^38^ Additionally, numerous centers refrain from implementing it due to the common occurrence of gastroesophageal dysfunction or diabetes, as well as the variability in the time of surgery. A 6-month individualized nutritional support program in malnourished HF patients lowered one-year mortality and hospital readmissions.^39^ This intervention optimized macronutrient intake (protein 15%-20%, carbohydrates 50%-55%, fat 30%-35% of total energy) and adjusted fiber (>25 g/day), sodium (<5 g/day), and fat composition (saturated 7%-8%, monounsaturated 15%-20%, polyunsaturated 5%, omega-6 >2 g/day, omega-3 >200 mg/day). Comorbidities, appetite loss, and digestive issues (e.g., dysphagia, nausea, dyspepsia) were also addressed, and nutritional supplements were used when needed. In the case of sarcopenic obesity, management prioritizes fat loss by reducing caloric intake (target BMI <35 kg/m²) while preserving muscle mass with increasing protein intake. In select cases, weight loss medications (e.g., GLP-1 inhibitors) or bariatric surgery may be an option.^40^ Finally, while screening for and treating osteopenia is common before transplantation, vitamin D supplementation could also enhance the effectiveness of prehabilitation programs, as it has shown improvements in physical performance among frail individuals.^41^

##### Psychological support

While pre-transplant depression does not appear to impact prognosis,^42^ early post-transplant depression is associated with an increased risk of long-term transplant-related morbidity and mortality.^43^ This effect may stem from reduced engagement in prehabilitation programs. Accordingly, the ISHLT and the European Society of Organ Transplantation recommends psychoeducational interventions for patients exhibiting symptoms of anxiety or depression, such as cognitive behavioral therapy or optimization of medical therapy for psychiatric illnesses. Stress-reduction strategies (e.g., mindfulness) may alleviate anxiety.”^44^ In a recent network meta-analysis of 186 randomized trials including 15,684 adults undergoing major elective surgery,^45^ psychosocial components alone did not reduce complications or length of stay. By contrast, when combined with exercise or nutrition, psychosocial support contributed to the greatest improvements in clinically meaningful outcomes, including shorter length of stay, higher health-related quality of life, and enhanced physical recovery. Thus, psychosocial interventions should be viewed as complementary rather than stand-alone strategies, systematically included in multimodal prehabilitation to reinforce adherence and augment the efficacy of exercise and nutrition in frail transplant candidates.

##### Cognitive support

Evidence for cognitive rehabilitation within prehabilitation remains extremely limited, with only 4 randomized trials included in the recent McIsaac network meta-analysis and inconclusive results.^45^ Nevertheless, its potential role within multimodal prehabilitation deserves exploration. Supporting this rationale, a randomized study in LTx recipients without prior impairment but with postoperative cognitive dysfunction showed that home-based Computerized Cognitive Training improved certain cognitive functions,^46^ suggesting that similar interventions could be evaluated in the pretransplant setting to address cognitive dysfunction.^47^

#### Anticipating and deterring postoperative frailty

Beyond the core drivers of frailty, several perioperative conditions can precipitate new or worsened frailty after transplantation. Anemia, and gastrointestinal and nutritional dysfunction contribute to perioperative vulnerability that exacerbate postoperative frailty. Primarily, these issues are confounded with the frail condition given their clinical symptoms of fatigue, weakness, malnutrition, shortness of breath, early satiety/aspiration. Evidence directly linking their management to frailty reversal is limited; therefore, we present them as anticipatory targets for pre- and perioperative risk mitigation.

##### Anemia

Anemia is often overlooked in the context of end-stage cardiopulmonary disease, as it is assumed to be a marker of chronic disease and inflammation. However, anemia itself carries prognostic significance: it increases peri-procedural transfusion risk, contributes to fatigue and functional decline, and may blunt the effectiveness of prehabilitation. Iron deficiency, with or without anemia, can aggravate hypoxic pulmonary hypertension in chronic lung disease,^48^ and directly impairs skeletal muscle metabolism, thereby reducing exercise tolerance. Evidence from cardiac surgery populations show that correction of anemia through intravenous iron, erythropoietin, repleting vitamin stores, and multimodal patient blood management is associated not only with reduced transfusion requirements, shorter length of stay, and even reduced mortality.^49^ In HF, multiple IV iron infusions improved 6MWT and quality of life, including in non-anemic iron deficiency.^50^, ^51^ These studies support iron repletion as a cornerstone of modern perioperative care, particularly in frail candidates where gains in functional capacity may translate into improved outcomes. For transplant candidates undergoing cardiopulmonary bypass or extracorporeal membrane oxygenation where anticoagulation is planned, occult bleeding (especially gastrointestinal) should be ruled out as a potential cause of anemia. In patients unresponsive to oral iron, intravenous administration is preferred due to impaired intestinal absorption from elevated hepcidin.^42^ While iron supplementation is generally safe, it should be used cautiously in patients with chronic infection. Although, in studies evaluating perioperative use, erythropoietin has not been associated with an excess thromboembolic risk comparable to that reported with long-term use, no data are available in patients awaiting lung transplantation, who may have chronic hypoxemia and a pro-inflammatory, procoagulant milieu.

##### Laryngopharyngeal and gastroesophageal dysfunction

Laryngopharyngeal dysfunction (LPD) and gastroesophageal dysfunction (GERD) are postoperative complications that threaten swallowing safety and intake, particularly frequent after HTx-LTx due to perioperative mechanical trauma/traction to adjacent aerodigestive structures and potential disruption of vagal and recurrent laryngeal nerve innervation, instrumentation (e.g., prolonged intubation, feeding tubes, and transesophageal echocardiography) and polypharmacy-associated gastrointestinal dysmotility (e.g., nausea/vomiting or ileus related to immunosuppressants, opioids, and antibiotics). If unmanaged, it can perpetuate or worsen postoperative frailty via aspiration and malnutrition. Malnutrition is a significant contributor to patient frailty and, consequently, their resilience to overcome perioperative insults and complications. Following transplantation, LPD and GERD are common complications and can further exacerbate nutritional risk. Therefore, it is essential to optimize their treatment and, if possible, their prevention.

*LPD* encompasses swallowing abnormalities (dysphagia) and vocal cord dysfunction, which can lead to dysphonia and impaired airway protection. Its incidence is notably high following thoracic transplantation, with reported dysphagia rates reaching up to 70%. The underlying pathophysiology involves compromised respiratory function, prolonged intubation, recurrent laryngeal nerve damage, ICU-acquired weakness and chronic gastroesophageal reflux (GERD). Preoperative risk factors are GERD, low BMI, and advanced age, conditions frequently observed in frail patients. In elderly patients, swallowing rehabilitation has been shown to enhance safe oral intake, improve nutritional status, and reduce pneumonia rates. Moreover, a randomized controlled trial demonstrated that swallowing rehabilitative exercises were more effective than compensatory methods in improving clinical swallowing function and overall quality of life.^52^ Such strategies have not yet been evaluated in the context of HTx-LTx; therefore, whether incorporating these exercises into prehabilitation programs can help mitigate nutritional risk and reduce the likelihood of postoperative aspiration remains to be investigated.

*GERD* includes GERD, esophageal dysmotility, and gastroparesis. GERD is common in patients with end-stage lung disease, with studies showing a prevalence of 68%-88% particularly in LTx patients, and is associated with adverse pulmonary outcome through repeated micro-aspiration. Because symptoms are unreliable indicators, targeted pH-impedance testing may be considered in LTx candidates at high risk of micro-aspiration, aiming is to protect swallowing safety and nutritional status while also contributing to improved graft function.^53^ Surgical treatment seems to be more effective in preventing graft dysfunction than the medical treatment alone,^54^ especially when performed early,^55^ but this practice remains controversial. Recent evidence suggests that some minimally invasive approaches to reflux may be feasible and beneficial.^55^

## Future directions

A key challenge for all transplant providers is applying a consistent measure of frailty during evaluation of thoracic transplant candidates. More complex measures of body composition in combination with biomarker testing may be more comprehensive and predictive than BMI for assessing sarcopenia and frailty,^25^ especially in cardiopulmonary failure patients. In the context of LTx, building off the LT-FS tool to measure frailty, Singer et al propose 2 enhanced versions of the tool that improve its prediction of morbidity and mortality: one incorporating body composition (using appendicular skeletal muscle index and body fat by bioelectrical impedance analysis), and another combining body composition with biomarkers. Both models demonstrated improved performance in categorizing patients into frail and non-frail states and predicting morbidity and death. Similarly, a *Heart Failure Frailty Score* was established through expert consensus in 2025.^56^ The development of new biomarkers will stem from a deeper understanding of the pathophysiological processes underlying frailty, including chronic inflammation, cellular senescence, mitochondrial dysfunction, and impaired nutrient sensing. These biomarkers could play a key role in distinguishing the reversible aspects of frailty and include cellular senescence biomarkers (e.g., senescence-associated β-galactosidase), DNA damage markers (e.g., p53), cell cycle inhibitors (e.g., p16), and gene panels identifying senescent cells. Multicenter randomized trials that evaluate prehabilitation, nutrition, and exercise programs are needed to better understand the trajectory of the endotype for the frailty phenotype among heart and lung failure patients. Improvements in risk stratification through biomarker validation of endotyping could help avoid transplanting patients for whom surgery would not reverse frailty but precipitate worsening morbidity and mortality. In addition, biomarkers of biological aging (e.g., epigenetic clocks and molecular aging signatures) have been proposed to complement functional testing and conventional biomarkers for longitudinal monitoring; however, their utility as surrogates for frailty remains uncertain in lung transplantation given the heterogeneity of aging syndromes. Indeed, recent data suggest that frailty is more closely associated with telomere dysfunction than with epigenetic aging.^57^, ^58^

While recent data strongly support the effectiveness of prehabilitation programs in transplant candidates, the optimal interventions remain undefined, particularly when it comes to nutrition. Several other approaches remain to be explored, such as the Mediterranean diet, nutritional hormetins (e.g., polyphenols and flavonoids), and immunonutrition formulas enriched with arginine, omega-3 fatty acids, and ribonucleotides, which have shown to reduce infection and hospital stay after carcinologic surgeries.^59^ Future research should also investigate whether prehabilitation should be systematically adapted to the underlying pathology (HF vs end-stage lung disease). While current programs individualize primarily according to baseline functional performance and disease severity, no study has yet compared disease-specific adaptations. Such work could clarify whether tailoring interventions to organ-specific pathophysiology improves outcomes beyond the established benefits of general prehabilitation. Furthermore, consistently measuring and enhancing adherence to prehabilitation programs is critical, as frail patients who stand to benefit the most are often the least likely to engage. Regarding mechanical circulatory support, while frail patients receiving left ventricular assist devices face higher long-term mortality, survivors might experience reversal of frailty, highlighting frailty as a dynamic condition and reinforcing the need to refine patient selection criteria.^60^ Another important avenue for future research is the role of prehabilitation response in guiding transplant decisions. A lack of improvement despite adequate and well-conducted prehabilitation may serve as an objective signal to re-evaluate transplant candidacy, though it should not be considered an absolute contraindication at this stage.

## Conclusion

Frailty is increasingly recognized as a critical determinant of outcomes in thoracic transplantation, particularly as the recipient profile continues to evolve toward older and more comorbid patients. While frailty assessment has become an integral part of transplant evaluation, its precise role in candidate selection remains complex due to the heterogeneity of frailty phenotypes and the absence of standardized assessment tools. However, the identification of frail or at-risk individuals provides an opportunity for targeted interventions aimed at optimizing physiological resilience before transplantation. Prehabilitation strategies encompassing physical exercise, nutritional optimization, and psychological support have shown promise in improving perioperative outcomes and mitigating the risks associated with frailty. Nevertheless, significant knowledge gaps remain, particularly regarding the reversibility of frailty, the most effective intervention modalities, and the potential role of novel biomarkers in refining risk stratification.

## Conflicts of Interest statement

The authors declare the following financial interests/personal relationships, which may be considered as potential competing interests: JM received congress reimbursement fees from Biotest/Grifols and Therakos. KG received research grant support to Duke from Octapharma and the Patient-Centered Outcomes Research Institution (PCORI). BB is UptoDate Reviewer. The other authors declare that they have no known competing financial interests or personal relationships that could have appeared to influence the work reported in this paper.

## Financial support

The authors received no specific funding for this work.

## Ethical Approval and Consent to Participate

Not applicable.

## Availability of Supporting Data

Not applicable.

## Authors Contributions

AP and BB wrote the first draft. All authors revised and approved the final manuscript.
